# Prevalence and Bacterial Isolates of Mastitis in Dairy Farms in Selected Districts of Eastern Harrarghe Zone, Eastern Ethiopia

**DOI:** 10.1155/2017/6498618

**Published:** 2017-03-02

**Authors:** Tesfaheywet Zeryehun, Gerema Abera

**Affiliations:** College of Veterinary Medicine, Haramaya University, P.O. Box 301, Haramaya, Ethiopia

## Abstract

The study was conducted from November 2015 to April 2016 to estimate the prevalence of clinical and subclinical mastitis in lactating cows, to assess the associated risk factors, and to isolate the major bacterial pathogens in dairy farms in selected district of Eastern Harrarghe Zone, Eastern Ethiopia. The study was carried out in 384 dairy cows based on data collection, farm visit, animal examination, California mastitis test (CMT), and isolation bacterial pathogens using standard techniques. In the present study the overall mastitis at cow level was 247 (64.3%). The prevalence of clinical and subclinical mastitis and quarter level prevalence for clinical and subclinical mastitis were 12.5% and 51.8% at cow level and 10.7% and 46.4% at quarter level, respectively. Clinically, 101 (6.6%) quarters which belong to 75 (19.5%) animals were found to be with blind teat. In the present study prevalence of mastitis was significantly associated with parity and age (*p* < 0.05). Bacteriological examination of milk sample revealed 187 isolates where* coagulase negative Staphylococcus* species (CNS) (34.2%) was the predominant species while* Streptococcus faecalis* (2.1%) was identified as the least bacteria. The present study concluded that prevalence of mastitis particularly the subclinical mastitis was major problem of dairy cows in the area and hence warrants serious attention.

## 1. Introduction

Ethiopia has the largest livestock population in Africa, where cows are among the huge number of the cattle population, where the milk harvested from these animals serves an important dietary source for most of the rural, urban, and periurban population [1]. Nonetheless, milk production from these animals is below its potential failing the demands of the population in the country. The factors that contributed to the poor performance of the dairy subsectors in the countries include but are not limited to poor productivity, inappropriate technology, poor infrastructures, and inadequate animal feed and animal health services [1, 2]. In Ethiopia the dairy sector is believed to contribute to the micro and macro economy by securing household nutrition and alleviating poverty in the country, respectively [3]. Nevertheless, the quality and quantity of milk in the country were tremendously declined due to various causes including mastitis.

Mastitis is a multietiologic disease of the mammary gland characterized mainly by reduction in milk production and considered an economically important disease in the dairy subsector in developed and developing nations [4, 5] including Ethiopia [6, 7]. Studies have indicated that it is among the major economically important diseases in most dairy farms in Ethiopia [8, 9]. Furthermore, mastitis could be a danger to human health because milk from mastitic udder of animal is contaminated with bacteria which could be potential source of infection to consumers [10] and many of them are responsible for diseases like tuberculosis, streptococcal intoxication, colibacillosis, streptococcal sore throat, and brucellosis in human [11].

Mastitis is universally classified as clinical and subclinical mastitis [12]. Clinical mastitis is characterized mainly by appearances of changes in the milk such as flakes and clots and presence of signs of inflammation on the mammary glands such as swelling, heat, pain, and edema [13–15], as well as systemic signs on the animal including fever, rapid pulse, appetite loss, dehydration, and depression [16]. Subclinical mastitis is that which is mainly characterized by absence of visible appearance of changes in the milk or udder, but milk production decreases, bacteria are present in the secretion, and composition is altered [13]. Hence, detection of the milk is not possible clinically but only by determining high somatic cell count (SCC) in milk or by bacterial culture [10].

Mastitis being a multietiologic disease, many microorganisms are implicated as causes. Majority of microorganisms that are responsible for mastitis and spoilage of milk are of bacterial origin and include* Staphylococcus aureus, Streptococcus agalactiae, Corynebacterium bovis, Mycoplasma* species,* Streptococcus uberis* [13], coliforms (*Escherichia coli, Klebsiella* species, and* Enterobacter aerogenes*),* Serratia, Pseudomonas, Proteus* species, environmental* Streptococci, and Enterobacter* species [10]. These organisms are usually found in the environment of the cow; hence they can easily be contracted by the udder [5, 10].

In Ethiopia a few studies have been conducted with the purpose of estimating prevalence of mastitis [17–23]; however, mastitis as a disease, particularly the subclinical mastitis, has received very little attention. Furthermore, control and prevention of such important disease in the dairy sector require a rigorous and systematic research and documentation of information on the status of the disease.

Therefore, the present study was aimed to estimate the prevalence of mastitis in lactating dairy cows, to assess the associated risk factors, and to isolate and identify the major bacterial pathogens from milk samples of mastitic cows.

## 2. Materials and Methods

### 2.1. Study Area Description

A cross-sectional study was conducted in the selected farms in eastern Hararghe including dairy farms in Haramaya district, Haramaya University, Awaday, Dire Dawa Administrative city, Addalle, Haramaya town, and Harar town. Haramaya district is located in the Eastern Harrarghe Zone of the Oromia Region of Ethiopia, which are about 506 kilometers from Addis Ababa and 12 kilometers far from the city of Harar and 35 kilometers from Dire Dawa and 5 kilometers from Haramaya University at an altitude of 2047 meters above sea level (m a.s.l.) between latitude 9°24′′N and longitude 42°01′′E. The mean annual rainfall is 870 mm with a range of 560 to 1260 mm and the mean maximum and minimum temperatures are 23.4°C and 8.25°C, respectively [24]. Harar town was located 525 km away from the capital city Addis Ababa at an altitude of 1300–2200 m a.s.l. between 42040′′ and 42022′′ east longitudes and 100 and 250 north latitude lines and it is a capital city of both eastern Hararghe zone and Harari regional state [25] and Dire Dawa administrative region which was located in eastern part of Ethiopia about 515 kms from Addis Ababa located approximately between latitudes 9°27′ and 9°49′N and longitudes 41°38′ and 42°19′E and lies in 950–1250 m a.s.l. The average maximum and minimum temperatures are 31.4°C and 18.2°C, respectively, and annual rain fall that the region gets from April and July is about 604 mm [26].

### 2.2. Study Population and Husbandry Practices

The target population was lactating Holstein-Zebu cross-breeds and local Zebu cows from 20 dairy farms from four districts in Eastern Harrarghe Zone, Eastern Ethiopia. The cow attributes including age in year (young adult 3–5, adult 6–10, and old ≥11), breed (cross and Zebu), parity (1–6), and stage of lactation in months (early 1–3, mid 4–6, and late ≥7) were recorded. 327 (85%) of the animals were kept indoors and are supplemented by products of beer, molasses, and hay and 14 (70%) of the farms were intensive production where dairy animals are kept indoors at zero grazing.

### 2.3. Sample Size and Sampling Methods

A total of 20 dairy farms comprising 3 from Haramaya districts (Haramaya town, Haramaya University, Addalle); 1 from Awaday, 7 from Harar town, and 9 from Dire Dawa were selected based on accessibility and willingness of owners. The sample size was determined using the formula recommended by Thrusfield [27]:

(1) $$\begin{array}{c} n=\frac{{\left(1.96\right)}^{2}P_{exp}\left(1-P_{exp}\right)}{d^{2}}. \end{array}$$

In this formula, *n* is the required sample size, 1.96 is the value of *Z* at the 95% confidence level, *P*_exp_ is the expected prevalence of mastitis, and *d* is the desired absolute precision which is 5%. 384 lactating cows were randomly sampled using simple random sampling method with the expected prevalence of 50% from 20 selected dairy farms and small holders out of more than 75 dairy farms dairy cows in study area.

### 2.4. Protocol Design and Method

In this study physical examination of the udder, California mastitis test and bacterial isolation were conducted following standard procedures. Briefly, udders or teats were physically examined first by visualization and then by palpation to detect the presence of gross lesion. Clinical mastitis was diagnosed on the basis of manifestation of visible signs of inflammation and abnormal milk. A quarter, which was warm and swollen and had pain upon palpation, were considered to have acute clinical mastitis. Viscosity and appearance of the milk secretion from each quarter were examined for the presence of clots, flakes, blood, and watery secretion. Besides, rectal temperature was taken for acute mastitis cases to check systemic involvement of the infection. On the other hand, atrophied, misshaped, and any blind, hard, and fibrotic quarters were considered to have chronic mastitis [10, 11].

California mastitis test (CMT) was carried out following the procedure described by NMC [28] and Quinn et al. [10] for screening subclinical mastitis. Briefly, a drop of the CMT reagent (4% NaOH in distilled water and 1% bromothymol blue) was put on the 4 cups of the CMT paddle into which equal amount of milk from the respective quarters of the cow was added and gently mixed by rotating the paddle in a horizontal plane for 20–30 seconds. The test result was interpreted based on the thickness of the gel formed by CMT reagent and milk mixture as 0 and trace for negative and +1, +2, and +3 for positive. Cows were considered positive for CMT, when at least one-quarter turned out positive. A herd was considered positive for CMT, when at least one cow in a herd is tested positive with CMT. The total number of blind teats as well as those with clinical infection was subtracted from the total number of teats and the difference was used to calculate the prevalence of subclinical mastitis.

Bacterial culture and identification were conducted aseptically and collected by standard milk sampling techniques [28]. First, after the hands were cleaned by detergents and clean water, the udder and teats were washed with tap water and dried. If there is a considerable amount of dirt, it was properly removed. The teat on the far side of the udder is cleaned first and then those on the near side [29]. Then the teat ends were vigorously swabbed with cotton soaked in 70% ethyl alcohol prior to sampling. Approximately 10 mL of milk was collected from clinical and subclinical (CMT positive) mastitic cows into horizontally held sterile test tube after discarding the first 2-3 milking streams. The samples were placed in racks for ease of handling and transported in an icebox to the Microbiology Laboratory of College of Veterinary Medicine in Haramaya University, where they were stored at 4°C for a maximum of 24 hrs until inoculation on a standard bacteriological media was done [28].

Bacteriological examination was done according to the NMC [28], Quinn et al. [10], and National Committee for Clinical Laboratory Standards (NCCLS) [30]. Refrigerated milk samples were warm at room temperature (25°C) for about an hour and then mixed by shaking in order to disperse bacteria and fat. Samples were allowed to stand for a while for foam to disperse before just inoculation. A loopful of milk sample was streaked on tryptose blood agar base enriched with 7% defibrinated sheep blood (Oxoid, UK) using the quadrant streaking method for each quarter. Blood agar plates were incubated aerobically at 37°C. The plates were checked for growth after 24 hr, 48 hr, and 72 hr to rule out slow growing bacteria. The plates were examined for growth, morphological features, such as colony size, shape, and color, and hemolytic characteristic. Suspected colonies were subcultured on nutrient agar plate (Oxoid, UK) for further investigation.

After pure colonies were obtained, Gram stained smears were done for primary identification of bacteria to genus level, such as Gram reaction (Gram positive and Gram negative), and cellular morphology (coccus or rods). Other primary tests done include catalase, oxidase, motility, and coagulase tests and growth or absence of growth on MacConkey agar (Oxoid, UK),* Bacillus cereus* agar (Oxoid, UK), Mannitol salt agar (Oxoid, UK), and Edward medium agar (Oxoid, UK). For secondary identification of the isolates to the species level, different biochemical tests (oxidation fermentation test, Mannitol and salicin sugar fermentation, aesculin hydrolysis, and CAMP test) were done based on the genus of bacteria to be identified [10].

### 2.5. Statistical Analyses

The data collected during the study periods were entered into MS Excel spread sheet and analyzed using SPSS software (SPSS version 20). The effect of risk factors with possible association of the disease was analyzed using Chi-square. The associations between dependent and independent variables were tested, and *p* < 0.05 was taken as statistically significant [31].

## 3. Results and Discussion

### 3.1. Results

*Prevalence at Cow and Quarter Level.* In the present study the overall prevalence of mastitis was 64.3% (247/384) at cow level where 12.5% (48/384) and 51.8% (199/384) cows were found with clinical and subclinical mastitis, respectively (Table 1). Out of the 1536 quarters examined, 101 (6.6%) quarters which belong to 75 (19.5%) animals were with blind teat. Upon screening of the functional teats (1536) by CMT, 713 (46.4%) quarters were found to be affected by subclinical mastitis and 164 (10.7%) by clinical mastitis. In quarter prevalence of subclinical mastitis, right hind (RH) teats showed the highest ratio of infection (50.5%) followed by the left hind (LH) (43.9%). The overall quarter prevalence of subclinical mastitis was 41.4% (Table 2). 

*Prevalence of Mastitis on the Basis of Location.* The prevalence of mastitis from dairy cows that originated from different locations was compared (Table 3). In the present study the heist prevalence of mastitis was recorded from Dire Dawa, while the lowest was found in cows that originated from Awaday area. However, there was no significant association between prevalence and origin of animals (*p* > 0.05). 

*Prevalence with respect to Risk Factors.* Prevalence of mastitis related to specific risk factors was determined as the proportion of affected cows out of the total examined. Age and parity were found to be having significant difference on the prevalence of bovine mastitis (*p* < 0.05) as indicated in Table 4. Cows at age group of young adult, adult, and old had prevalence of 31.4%, 66.7%, and 58.3%, respectively. Higher prevalence (69.8%) was recorded in cow giving birth to three calves followed by cow giving birth to six calves (62.5%) as compared to cows having another number (1, 2, 4, and 5) of calves (Table 4). 

*Bacterial Isolation.* Milk samples of 594 quarters, which were positive for CMT from 247 (64.3%) cows, 48 (12.5%) clinical and 199 (51.9%) subclinical mastitic cows, were cultured for microbiological examination and yielded 187 (31.5%) bacteria. The bacterial isolation rate and their prevalence are shown in Table 5. The predominant isolated bacteria were* coagulase negative Staphylococcus* species (CNS) with isolation rate of 34.2% followed by* Staphylococcus aureus* with isolation rate of 24.2%.* Streptococcus agalactiae* was the third predominant isolated bacteria with isolation rate of 17.1%.* Streptococcus faecalis* was the least isolate which accounts for 2.1%.

### 3.2. Discussion

In the present study the overall prevalence of mastitis was 64.3%. The result was in agreement with Nibret et al. [29] and Mekonnen et al. [32] who reported prevalence of 60.9 and 62.9%, respectively, and closely in agreement with the finding of 71% around Holeta Town [19], 59.1% in Borena [33], 56.5% in Batu and its surrounding [34], and 56.16% in West Algeria [35]. But the study is in disagreement with findings of 75.22% in Jimma Town [21], 74.3% in Addis Ababa area [22], 53.25% in Dire Dawa town [36], 52.78% in and around Sebeta [37], 46.7% in Adama town [38], 44.1% around Holeta areas [39], 32.6% in and around Gondar [29], 28.2% in Bahir Dar and its surroundings [40], and 34.9% in Southern Ethiopia [41]. The discrepancies in these studies could be attributed to the difference in the breed, management system, and the epidemiological status [11].

The present study also showed prevalence of 10.7% for clinical mastitis that was closer to the reports of 9.09% in Dire Dawa town [36], 10.0% in Adama town [38], 10.3% at Asella [42], 10.3% around Holeta Town [39], and 11.9% in Bahir Dar and its surroundings [40]. However, the present finding was much higher than the findings of 0.93% in and around Gondar [29], 5.3% in Batu and its surroundings [34] and much lower than the reports of 22.4% around Holeta Town [19], 19.6% in Addis Ababa [22], and 16.11% in and around Sebeta [37]. Risk factors which influence the occurrence of clinical mastitis were outlined as animal, pathogen, and environmental risk factors, which could contribute in the discrepancies of mastitis prevalence [11].

The study also revealed subclinical mastitis prevalence of 51.8% which agrees with the finding of 55.1% in Addis Ababa [22], 55.8% in Asella [20], 44.6% around Holeta Town [19], and 44.16% in Dire Dawa town [36]. But the subclinical mastitis recorded in the present study was in closer agreement with previous findings such as the findings of 40.6% in Batu and its surroundings [34], 33.8% around Holeta areas [39], 36.67% in and around Sebeta [37], 31.67% in and around Gondar [29], and 23.0% in Bahir Dar and its environments [40]. Since environmental factors play significant role, the prevalence of subclinical mastitis varies in dairy animals [11].

The present study revealed higher prevalence of subclinical mastitis compared to clinical mastitis. Other studies shared similar observations [21, 22, 43, 44]. This could be attributed to the invisible and silent nature of subclinical mastitis which is usually given little attention by farms when it comes to treatment unlike clinical mastitis towards which treatment and control efforts are concentrated [39, 45].

The quarter level prevalence of mastitis in the present study was 57.1% where 10.7% were clinical and 46.4% were subclinical. So the study result is closer to the findings of 62.3% at Addis Ababa [22] and 47.52% at Addis Ababa and Sebeta Town [46]. Similarly, Mekibib et al. [19] reported an overall prevalence of 44.9% around Holeta Town, where 10% and 34.8% represent prevalence of clinical and subclinical mastitis. On the contrary the present finding was much higher than the prevalence of 30.32% reported by Biniam et al. [36] in Dire Dawa town, where 9.68% were clinical and 20.65% were subclinical. It was also higher than reports made overseas such as prevalence of 35.25% in Pakistan [47] and 27.57% in Germany [48]. The difference may be due to greater experience in drying off, the potential effect of level of milking hygiene, herd size and cleanness, and the application of sanitary measures in these farms. With regard to prevalence of mastitis in each quarter of the udder, the right hind quarters were affected with the highest infection rate 50.5%. The left hind quarters were the second with an infection rate of 43.9%. Zeryehun et al. [22] attributed the highest infection rates in these quarters to the high production capacity of the rear quarters and the high chance of getting fecal and environmental contamination.

The study showed that there were significant statistical associations (*p* < 0.05) between the prevalence of mastitis with the age and parity of animals, where risk of mastitis increases with age and parity number. The present result was in agreement with the observation of Nibret et al. [29] who stated that parity and age are significantly associated with infection rates. Similarly Girma [39] in Holeta area and [40] around Bahir Dar area have reported that cows with many number of cows were with higher prevalence of mastitis, while similar to the finding of the present study, prevalence of mastitis was reported to increase with age in study conducted in Khartoum, Sudan [49], and around Holeta Town [19]. The higher prevalence in older cows in the present study might be that older cows have largest teats and more relaxed sphincter muscles that render ease of accessibility and establishment of infectious agent in the cows' udder [11].

The current study showed statistically insignificant prevalence of mastitis among different breeds, and lactation stages which is = in agreement with previous studies conducted elsewhere [29, 34, 37, 40, 49]. Although insignificant, the prevalence of mastitis was relatively higher in early and mid lactation compared to the late lactation; this might be associated with delayed diapedesis of neutrophils into the mammary gland [50].

The present study resulted in isolation of numerous pathogenic bacteria. The most dominant pathogenic species that causes clinical and subclinical mastitis in the study area were* coagulase negative Staphylococcus* (34.2%) followed by* S. aureus* (24.1%). The predominance of* coagulase negative Staphylococcus* (43.47%) and* S. aureus* (36.95%) was also reported in a study conducted elsewhere in the country [19, 21, 23, 40, 43]. In this study the least isolated bacteria were* C. bovis, S. uberis, B. cereus, and Micrococcus* spp. which is in agreement with reports of Sori et al. [21].

## 4. Conclusions

The present study recorded an overall prevalence of 64.3%, which might entail that mastitis was a major health problem of dairy cows which undoubtedly will have drawback on productivity of dairy industry and hence warrants serious attention. Particularly the prevalence of subclinical mastitis was high in the study areas (51.8%) which might mean dairy farm managers are only concerned with clinical form of mastitis and often are unawareness of the status of subclinical infection in the herd. The present study identified several bacterial species the highest of which was* coagulase negative Staphylococcus*. Hence regular screening for the detection of subclinical mastitis and proper treatment of the clinical cases as well as appropriate treatment of cows during dry and lactation period should be practiced.

## Figures and Tables

**Table 1:** Prevalence of clinical and subclinical mastitis at cow and quarter levels.

Form of mastitis	Total examined cows	Total number affected (%)	Total examined quarter	Total number affected (%)
Clinical	384	48 (12.5)	198	164 (10.7)
Subclinical	384	199 (51.8)	791	713 (46.4)

*Total*	*384*	*247 (64.3)*	*989*	*877 (57.1)*

**Table 2:** Quarter prevalence of subclinical mastitis using California mastitis test.

Quarter	Not examined	Positive	Frequency (%)
LF	351	115	32.8
LH	360	158	43.9
RF	348	131	37.6
RH	376	190	50.5

*Total*	*1435*	*594*	*41.4*

**Table 3:** Prevalence of bovine mastitis at place of origin.

Origin	Total number of cows examined	Mastitis positive (prevalence %)			*χ* ^2^	*p* value
		Clinical	Subclinical	Total		
Awaday	26	6 (23.1)	14 (53.8)	20 (76.9)	4.170	0.244
Harar	105	7 (6.7)	63 (60)	70 (66.7)		
Haramaya	91	26 (28.6)	44 (48.3)	70 (76.9)		
Dire Dawa	162	9 (5.6)	78 (48.1)	87 (53.7)		

*Total *	*384*	*48 (12.5)*	*199 (51.9)*	*247 (64.3)*		

**Table 4:** The prevalence of both clinical and subclinical mastitis in milking cows based on age, stage of lactation, parity number, and breed.

Host risk factors	Total number of animals examined	Number of animals affected	Prevalence (%)	*χ* ^2^	*p* value
*Age*					
Young adult	153	48	31.4	43.449	0.000
Adult	195	130	66.7		
Old	36	21	58.3		

*Lactation*					
Early	152	77	50.7	3.808	0.149
Mid	80	49	61.3		
Late	152	73	48.0		

*Parity number*					
1 calf	126	40	31.7	32.428	0.000
2 calves	101	58	57.4		
3 calves	53	37	69.8		
4 calves	59	36	61.0		
5 calves	37	23	62.2		
6 calves	8	5	62.5		

*Breed*					
Cross	327	170	52.0	0.024	0.877
Local	57	29	50.9		

**Table 5:** Bacterial species isolated from both clinical and subclinical mastitic cows.

Bacterial species	Clinical (%)	Subclinical (%)	Total number of isolates	Prevalence (%)
*Coagulase negative Staphylococcus *	2 (3)	62 (97)	64	34.2
*Staphylococcus aureus*	32 (71)	13 (29)	45	24.1
*Streptococcus agalactiae*	2 (6)	30 (94)	32	17.1
*Streptococcus dysgalactiae*	12 (100)	0	12	6.4
*Micrococcus species*	0	12 (100)	12	6.4
*Streptococcus uberis*	0	7 (100)	7	3.7
*Corynebacterium bovis*	0	6 (100)	6	3.3
*Bacillus cereus*	0	5 (100)	5	2.7
*Streptococcus faecalis*	1 (25)	3 (75)	4	2.1

*Total *	*49 (26)*	*138 (74)*	*187*	*100*

## References

[1] CSA (Central Statistical Agency) (2010). *Livestock and Livestock Characteristics, Agricultural Sample Survey*.

[2] Williams O., DeRosa D. A., Badiane O. Macroeconomic, international trade and sectoral policies in livestock development. An analysis with particular reference to low income countries.

[3] Mohamed A. M., Ehui A. S., Assefa Y. (2006). Dairy development in Ethiopia. *EPTD Discussion Paper*.

[4] Korhonen H., Kaartinen L., Sandholm M., Honkanen T., Buzalski L., Kaartinen, Pyörälä S. (1995). Changes in the composition of milk induced by mastits. *The Bovine Udder and Mastitis*.

[5] Bradley A. J. (2002). Bovine mastitis: an evolving disease. *Veterinary Journal*.

[6] Fekadu K. Survey on the prevalence of bovine mastitis and the predominant causative agents in Chaffa valley.

[7] Mekonnen H. M., Asmamaw K., Courreau J. F. (2006). Husbandry practices and health in small holder dairy farms near Addis Ababa, Ethiopia. *Preventive Veterinary Medicine*.

[8] Lema M., Kassa T., Tegegne A. (2001). Clinically manifested major health problems of crossbred dairy herds in urban and periurban production systems in the central highlands of Ethiopia. *Tropical Animal Health and Production*.

[9] Mungube E. O. (2001). *anagement and economics of dairy cow mastitis in the urban and periurban areas of Addis Ababa (Addis Ababa milk shed) [M.S. thesis]*.

[10] Quinn P. J., Carter M. E., Markey B. K., Carter G. R. (2002). *Clinical Veterinary Microbiology*.

[11] Radostits O. M., Gay K. W., Hinchcliff C. C., Constable P. D. (2007). Mastitis. *Veterinary Medicine: A Text Book of Disease of Cattle, Sheep, Pigs, Goats, and Horses*.

[12] Mungube E. O., Tenhagen B.-A., Kassa T. (2004). Risk factors for dairy cow mastitis in the central highlands of Ethiopia. *Tropical Animal Health and Production*.

[13] Erskine R. J., Bartlett P. C., Johnson G. L., Halbert L. W. (1996). Intramuscular administration of ceftiofur sodium versus intramammary infusion of penicillin/novobiocin for treatment of Streptococcus agalactiae mastitis in dairy cows. *Journal of the American Veterinary Medical Association*.

[14] Blowy R., Edmondson P. (2010). Introduction. *Mastitis Control in Dairy Herds*.

[15] Christos M. (2011). *Study on the prevalence and risk factors of bovine mastitis in and around Mekelle small scale dairy farms [DVM thesis]*.

[16] Schroeder J. W. (2010). *Mastitis Control Program. Bovine Mastitis and Milking Management*.

[17] Kifle A., Tadele T. (2008). Prevalence of sub clinical mastitis in small holder dairy farms in Selale, North Shewa Zone, Central Ethiopia. *The Internet Journal of Veterinary Medicine*.

[18] Almaw G., Molla W., Melaku A. (2009). Prevalence of bovine subclinical mastitis in Gondar town and surrounding areas, Ethiopia. *Livestock Research for Rural Development*.

[19] Mekibib B., Furgasa M., Abunna F., Megersa B., Regassa A. (2010). Bovine mastitis: prevalence, risk factors and major pathogens in dairy farms of holeta town, central Ethiopia. *Veterinary World*.

[20] Bedada B. A., Hiko A. (2011). Mastitis and antimicrobial susceptibility test at Asella, Oromia Regional state. Ethiopia. *Journal of Microbiology Antimicrobials*.

[21] Sori T., Hussien J., Bitew M. (2011). Prevalence and susceptibility assay of *Staphylococcus aureus* isolated from bovine mastitis in dairy farms of Jimma town, South West Ethiopia. *Journal of Animal and Veterinary Advances*.

[22] Zeryehun T., Aya T., Bayecha R. (2013). Study on prevalence, bacterial pathogens and associated risk factors of bovine mastitis in small holder dairy farms in and around addis Ababa, Ethiopia. *Journal of Animal and Plant Sciences*.

[23] Dabash H., Petros A., Fekadu A. (2014). Prevalence and identification of bacterial pathogens causing bovine mastitis from crossbred of dairy cows in North Showa Zone of Ethiopia. *Global Veterinaria*.

[24] HADB (Haramaya Woreda Agricultural Development Bureau) (2015). *Annual Progress and Planning Report Format Document*.

[25] CSA (Central Statistical Agency) (2008). *Summary and Statistical Report of the 2007 Population and Housing Census*.

[26] Vacnada (2011). Dire Dawa administrative official data. *Project Progress Report Dire Dawa, Ethiopia*.

[27] Thrusfield M. (2005). *Veterinary Epidemiology*.

[28] NMC (National Mastitis Council) (1990). *Microbiological Procedures for the Diagnosis of Bovine Udder Infection*.

[29] Nibret M., Yilikal A., Kelay B. (2011). A cross sectional study on the prevalence of sub clinical mastitis and associated risk factors in and around Gondar, Northern Ethiopia. *International Journal of Animal and Veterinary Advances*.

[30] National Committee for Clinical Laboratory Standards (NCCLS) (1999). *Performance Standards for Antimicrobial Susceptibility Testing. Ninth Informational Supplement*.

[31] Snedecor G. W., Cochran W. G. (1989). *Statistical Methods*.

[32] Mekonnen L., Tesfu K., Azage T. (2012). Clinically manifested major health problem of cross breed dairy herds in urban and peri-urban production system in central highlands of Ethiopia. *Tropical Animal Health and Production*.

[33] Bedane A., Kasim G., Yohannis T., Habtamu T., Asseged B., Demelash B. (2012). Study on prevalence and risk factors of bovine mastitis in Borana pastoral and agro-pastoral settings of Yabello District, Borana Zone, and Southern Ethiopia. *American-Eurasian Journal of Agricultural and Environmental Science*.

[34] Bedacha B. W., Mengistu H. T. (2011). Study on prevalence of mastitis and its associated risk factors in lactating dairy cows in Batu and its environments, Ethiopia. *Global Veterinaria*.

[35] Benhamed N., Moulay M., Aggad H., Henni J. E., Kihal M. (2011). Prevalence of mastitis infection and identification of causing bacteria in cattle in the oran region West Algeria. *Journal of Animal and Veterinary Advances*.

[36] Biniam T., Rediet T., Yonus A. (2015). Prevalence and potential risk factors of bovine mastitis in selected dairy farms of Dire Dawa Town, Eastern Ethiopia. *Applied Journal of Hygiene*.

[37] Sori H., Zerihun A., Abdicho S. (2005). Dairy cattle mastitis in and around Sebeta, Ethiopia. *International Journal of Applied Research and Veterinary Medicine*.

[38] Abera M., Demie B., Aragaw K., Regassa F., Regassa A. (2010). Isolation and identification of *Staphylococcus aureus* from bovine mastitic milk and their drug resistance patterns in Adama Town, Ethiopia. *Journal of Veterinary Medicine and Animal Health*.

[39] Girma D. (2010). Study on prevalence of dairy cows around Holeta Areas, West Shewa Zone of Oromia Region, Ethiopia. *Global Veternaria*.

[40] Bitew M., Tafere A., Tolossa T. (2010). Study on bovine mastitis in dairy farms of Bahir Dar and its environments. *Journal of Veterinary and Animal Advances*.

[41] Biffa D., Debela E., Beyene F. (2005). Prevalence and risk factors of mastitis in lactating dairy cows in Southern Ethiopia. *International Journal of Applied Research and Veterinary Medicine*.

[42] Lakew M., Tolosa T., Tigre W. (2009). Prevalence and major bacterial causes of bovine mastitis in Asella, South Eastern Ethiopia. *Tropical Animal Health and Production*.

[43] Workineh S., Bayleyegn M., Mekonnen H., Potgieter L. N. D. (2002). Prevalence and aetiology of Mastitis in cows from two major Ethiopian dairies. *Tropical Animal Health and Production*.

[44] Dego O. K., Tareke F. (2003). Bovine mastitis in selected areas of southern Ethiopia. *Tropical Animal Health and Production*.

[45] Karimuribo E. D., Fitzpatrick J. L., Bell C. E. (2006). Clinical and subclinical mastitis in smallholder dairy farms in Tanzania: risk, intervention and knowledge transfer. *Preventive Veterinary Medicine*.

[46] Belay G. (2011). *Prevalence of bovine subclinical mastitis in dairy farms of Addis Ababa and Sebeta Town [DVM thesis]*.

[47] Bachaya H. A., Raza M. A., Murtaza S., Akbar I. U. R. (2011). Subclinical bovine mastitis in Muzaffar Garh district of Punjab (Pakistan). *Journal of Animal and Plant Sciences*.

[48] Fadlelmoula A., Fahr R. D., Anacker G., Swalve H. H. (2007). The management practices associated with prevalence and risk factors of mastitis in large scale dairy farms in Thuringia-Germany 1: environmental factors associated with prevalence of mastitis. *Australian Journal of Basic and Applied Sciences*.

[49] Madut N. A., Gadir A. E. A., Jalii I. M. E. (2009). Host determinants of bovine mastitis in semi- intensive production system of Khartoum State, Sudan. *Journal of Cell an Animal Biology*.

[50] Schalm D. W., Carroll E. J., Jain C. (1971). *Bovine Mastitis*.

